# EMO-MoviNet: Enhancing Action Recognition in Videos with EvoNorm, Mish Activation, and Optimal Frame Selection for Efficient Mobile Deployment

**DOI:** 10.3390/s23198106

**Published:** 2023-09-27

**Authors:** Tarique Hussain, Zulfiqar Ali Memon, Rizwan Qureshi, Tanvir Alam

**Affiliations:** 1 Fast School of Computing, National University of Computer and Emerging Sciences, Karachi Campus, Karachi 75030, Pakistan; 2 College of Science and Engineering, Hamad Bin Khalifa University, Doha 34110, Qatar

**Keywords:** mobile networks, video classification, action recognition, deep learning

## Abstract

The primary goal of this study is to develop a deep neural network for action recognition that enhances accuracy and minimizes computational costs. In this regard, we propose a modified EMO-MoviNet-A2* architecture that integrates Evolving Normalization (EvoNorm), Mish activation, and optimal frame selection to improve the accuracy and efficiency of action recognition tasks in videos. The asterisk notation indicates that this model also incorporates the stream buffer concept. The Mobile Video Network (MoviNet) is a member of the memory-efficient architectures discovered through Neural Architecture Search (NAS), which balances accuracy and efficiency by integrating spatial, temporal, and spatio-temporal operations. Our research implements the MoviNet model on the UCF101 and HMDB51 datasets, pre-trained on the kinetics dataset. Upon implementation on the UCF101 dataset, a generalization gap was observed, with the model performing better on the training set than on the testing set. To address this issue, we replaced batch normalization with EvoNorm, which unifies normalization and activation functions. Another area that required improvement was key-frame selection. We also developed a novel technique called Optimal Frame Selection (OFS) to identify key-frames within videos more effectively than random or densely frame selection methods. Combining OFS with Mish nonlinearity resulted in a 0.8–1% improvement in accuracy in our UCF101 20-classes experiment. The EMO-MoviNet-A2* model consumes 86% fewer FLOPs and approximately 90% fewer parameters on the UCF101 dataset, with a trade-off of 1–2% accuracy. Additionally, it achieves 5–7% higher accuracy on the HMDB51 dataset while requiring seven times fewer FLOPs and ten times fewer parameters compared to the reference model, Motion-Augmented RGB Stream (MARS).

## 1. Introduction

The proliferation of highly rich sensor mobile devices and social media has tremendously aggregated image and video datasets, resulting in numerous real-world applications, such as human action recognition, facial expression recognition, visual recognition, and video summarization, among others [1]. However, traditional hand-crafted features [2] are insufficient for classifying video content that contains complex and pervasive semantic information. Therefore, the state-of-the-art methods are based on deep learning, where the feature learning process is usually automated [3]. Deep learning-based architectures for video processing usually integrate the temporal information with convolutional neural networks (CNNs) [4].

Action recognition in videos is a complex computer vision task, which has attracted significant attention in recent years. This task involves recognizing human actions in video sequences and has applications in various domains, including surveillance, sports analysis, and human–robot interaction [5]. Traditional approaches to action recognition employed handcrafted features and classifiers [6], but convolutional neural networks (CNNs) have become the standard approach due to their ability to process grid-type data and learn local features. As video processing is computationally complex, the computer vision community also aims to develop lightweight action recognition models [7,8].

Efficient neural network architectures for action recognition, based on spatiotemporal features, seek to reduce computational costs while maintaining high accuracy. Examples of such architectures include the Two-Stream Inflated 3D ConvNet (I3D), which extends 2D CNNs to 3D by incorporating temporal information in the form of optical flow, and Temporal Segment Networks (TSN), which samples a few temporal segments from each video and aggregates their predictions. I3D has achieved cutting-edge results on various action recognition benchmarks while maintaining low computational costs, and TSN has achieved competitive results with significantly lower computational costs than 3D CNNs [9].

**Figure 1:**
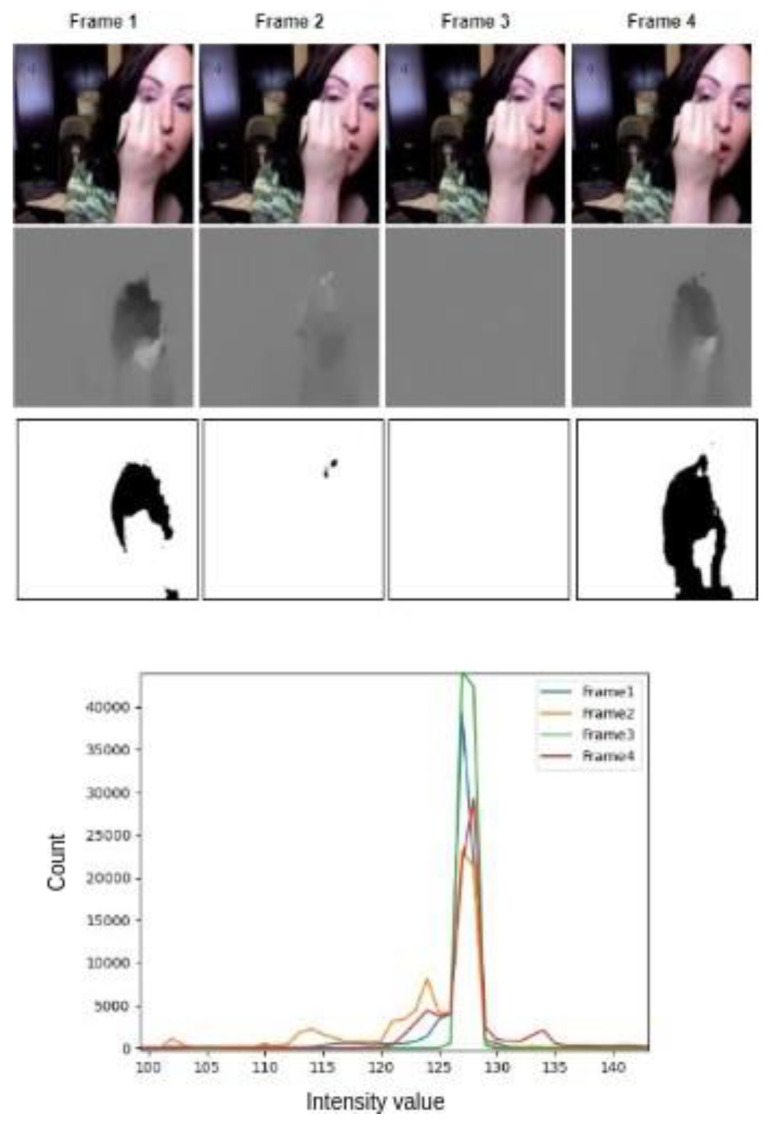
Optimal Frame Selection using the contextual information from the optical flow stream. It can be seen that some parts of the video can be more discriminative; frame 1 contains more information than frame 2 and frame 3, which can be observed from the histogram. The green graph is more stable than the other three frames because it has lower variance in the pixels as compared to the other frames.

Many studies [3,10] focused on randomly choosing frames for training. However, this has limitations. Some frames might not have enough useful information, and there can be repetitive frames. To address these issues, we use the Optical Flow method, which helps to understand how pixels move between frames, showing us how pixels change over time. In the visual representation depicted in Figure 1, it is evident that Frame 1 encompasses a substantial amount of information, whereas Frames 2 and 3 appear devoid of significant content. The visual disparity is indicated by the presence of black patches, denoting the magnitude of movement relative to the preceding frame. The application of Optical Flow facilitates the computation of pixel displacements across frames, allowing for the construction of histograms. This histogram analysis serves as a means to quantify the extent of pixel movement within each frame.

MoviNet [3] is a collection of lightweight neural network architectures designed specifically for real-time action recognition on mobile devices. It was created by Google researchers, based on the EfficientNet architecture, which reduces the computational cost, while maintaining high accuracy by combining depthwise separable convolutions and model scaling. It also employs temporal convolutions [11] to capture spatiotemporal features from video inputs. MoviNet models offer various efficiency and accuracy levels, ranging from MoviNet-Lite models for low-end devices to MoviNet models that achieve state-of-the-art accuracy on action recognition benchmarks. Overall, MoviNet models outperform other lightweight action recognition models in both accuracy and speed benchmarks. Additionally, the buffer stream process reduces memory usage and minimizes bottlenecks by processing videos in small consecutive sub-clips.

One limitation of the network is the dissipation of long-range temporal dependencies. To address this issue, the stream buffer concept has been introduced, storing feature maps of subclip boundaries to maintain temporal dependencies between consecutive non-overlapping subclips, thereby reducing memory usage. We further noticed the generalization gap [12], while implementing the MoviNet on the UCF101 [13] and HMDB51 dataset [14]. The model performed well with the training set but failed to perform well on the testing set. In neural networks, the distribution of network activation changes due to the changes in the parameters during training called the internal covariate shift (ICS) [15]. This can lead to hidden layers needing to adapt to new distributions, slowing down training and causing gradient optimization issues. Thus, we need to avoid this shift around to help gradient optimization and prevent vanishing gradients, and this will help run our training faster and reduce overfitting.

MoviNet utilizes batch normalization, which may be unstable with smaller batch sizes. Batch normalization normalizes activations during training using mini-batch statistics and allows dependencies between minibatch elements. This dependency can negatively impact model performance if the minibatch size is small or if elements do not exhibit parallelism. However, statistical estimation accuracy improves with larger batch sizes, although using a larger batch size can lead to higher generalization errors [16].

To address the dependency problem associated with batch normalization, it is necessary to consider normalization and activation functions together. Evolving Normalization (EvoNorm) [17] has been employed to overcome overfitting issues in this context. Unlike the other previous works [3,10], which dealt with normalization and activation separately, EvoNorm combines normalization and activation functions into a single tensor-to-tensor computation graph and evolves its structure starting from low-level primitives. Traditional activation functions rely on scalar-to-scalar transformations, while normalization utilizes mean subtraction and variance division. Given that these two functions often co-locate, training them separately may not be optimal.

In EvoNorm, the SILU/Swish [18] activation function is replaced with Mish [19] as follows.

(1) $$f\left(Bx\right)=\frac{x*\tanh\left(\ln\left(1+\exp\left(Bx\right)\right)\right)}{\sqrt{s_{\omega ,h,c}^{2}/g\left(x\right)}}\;$$

where *B* is a matrix and *x* is a vector. The $s_{\omega ,h,c}^{2}\;$ refers to the batch variance. we use ./*g* to indicate that aggregation is carried out in a grouped manner along a dimension.

This further improves the model’s performance. Mish is a non-monotonic activation function that has demonstrated superior performance compared to other activation functions in various deep-learning tasks. It has a smooth, continuous curve that enables better gradient flow and helps address vanishing gradient issues. By integrating EvoNorm with Mish, the model can potentially achieve faster training, reduce overfitting, and improve generalization performance on the testing set.

**Figure 2:**
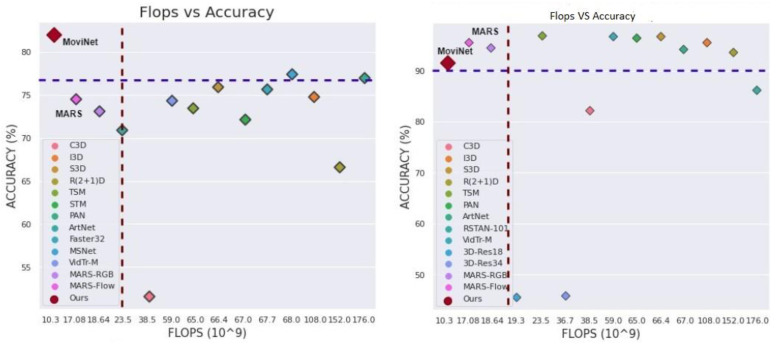
Flops vs. Accuracy: UCF101 (**Right**) and HMDB51 (**Left**). Here, MoviNet shows our proposed model (Red Diamond). In HMDB51, our model has outperformed all methods in both accuracy and computational complexity by a sufficient margin. Whereas, in UCF101, our model is giving fewer FLOPS and parameters at the cost of 12% accuracy.

Our research introduces the modified EMO-MoviNet-A2* architecture, which integrates EvoNorm, Mish activation, and optimal frame selection for improved action recognition accuracy and efficiency. This architecture also incorporates the stream buffer concept to maintain temporal dependencies and reduce memory usage. In Figure 2, we present a comparison of existing methods with the proposed strategy.

In summary, this research focuses on developing efficient neural network architectures for action recognition in videos, aiming to reduce computational costs while maintaining high accuracy. By employing advanced techniques such as depthwise separable convolutions, model scaling, and evolving normalization with Mish activation functions, these architectures can overcome the limitations of traditional models, like dependency issues and generalization gaps. The rest of this paper is organized as follows:

Section 2 and Section 3 presents related work, and proposed framework, respectively. Results and discussion are given in Section 4, and Section 5 concludes the paper.

## 2. Related Work

Convolution neural networks have been widely used for image-related tasks such as classification [20], object detection [21,22], and instance segmentation [23], and video classification [24]. Video classification entails analyzing video frames and identifying the objects, actions, scenes, and events in the video. This critical task finds applications in surveillance, sports analytics, content-based video retrieval, and video recommendation systems [25]. Recent advances in deep learning and computer vision have significantly improved video classification performance, making it an exciting and rapidly evolving field of study. However, several challenges associated with video classification still exist, such as the need for large amounts of labeled training data, high computational costs of processing video data, and the complexity of capturing a video’s temporal dynamics [26]. Long-short-term memory (LSTM)-based model [27], and the 3DCNN model [28] along with the temporal variation, is the optimal choice for video classification.

In this work, our focus is to build a computationally efficient model for action recognition in videos. We modified the recent work of Mobile Video Networks [3], and Motion Augmented RGB stream (MARs) [10]. We also focused on the optimal frame selection and generalization gap [29], which were the major shortcomings in the previous papers.

### 2.1. Computation and Memory Efficient Networks

In recent years, video classification has advanced tremendously since deep neural networks have remarkably enhanced. Many researchers have used 3D CNN [30,31] architecture with a temporal dimension along with a pre-trained 2D CNN model [32] to reduce the computation complexity and memory space. In recent works [33,34], improving the performance of video models has obtained extensive attention as model efficiency plays a vital role in real-world applications.

Squeeze Net [35] replaced 3 × 3 convolution with the 1 × 1 convolution to reduce the parameter, decrease the number of filters, and down sample in the network, so that the classification accuracy can be improved. Afterward, most of the work focused on reducing the number of operations and measuring latency. Particularly, MobilenetV1 [36] introduced the model, which not only deals with the spatial dimensions, but can also perform depth-wise separable convolutions, and which leads to fewer parameters by a great margin, as compared to the normal convolutions. Thus, MobilNetV1 dramatically reduces computational cost and model parameters. MovileNetV2 [37] is the improved version of [36], with two new features: Linear bottleneck between the layers and the shortcut connection between the bottlenecks. Shuffle Net [38] further reduced the number of parameters using the group convolution and channel shuffle operations. Condense Net [39] takes the benefit of dense connections learned by group convolutions during training to re-use features.

The Squeeze and Excitation (SE) [40] primarily focused on reducing the number of parameters. The SE block improves the quality of representations by modeling the inter-dependencies between the channels of the spatiotemporal features via a 3D average pooling operation.

### 2.2. Mobile Video Network (MoviNet)

MoviNet [3] is from the family of memory and search efficient architectures, which trade-offs the accuracy and efficiency, based on mixing spatial, temporal, and spatiotemporal operations. MoviNet also introduced the stream buffer, conducive to minimizing memory by processing on video in small consecutive sub-clips, that allows the model to run with sufficiently fewer memory bottlenecks. This also allows for a larger class of operations to improve temporal modeling. It has features from the previous MobileNet models, such as depthwise separable convolution [36], a Linear bottleneck between the layers, and the shortcut connection between the bottleneck [37]. We implemented this model on UCF101 and HMDB51 datasets and observed the overfitting issue, due to larger generalization error [41].

The recent research works [9,10,33] use batch normalization (BN). The BN has some major flaws; it normalizes the activations during training by using the mini-batch statistics, and it allows the dependency between the elements of the mini-batch. This dependency will attenuate the performance of the model if the minibatch size is smaller or if the elements do not follow the parallelism. However, several alternative methods [17] have been introduced to deal with these problems such as batch renormalization [42] and group normalization, but they still are not able to match the performance of BN for larger batch sizes and are still not able to solve the degradation problem during smaller batch size.

#### 2.2.1. Motion Emulating RGB Stream (MERS)

Motion Emulating RGB Stream (MERS) is used for generating flow characteristics from RGB input, by applying a loss function at the feature level. Low-level local features are represented by a CNN’s initial layers, whereas high-level global features are represented by its later layers, which are highly discriminative for the job at hand.

(2) $$LOSS_{MERS}=\|fc_{MERS}-fc_{FLOW}{\|}^{2}$$

where $fc_{MERS}$ and $fc_{FLOW}$ are features from MERS and FLOW, respectively. This loss is used to train the network.

#### 2.2.2. Motion-Augmented RGB Stream (MARS)

To further enhance the training with the appearance information, a combination of MSE and cross-entropy losses is used throughout the whole network. MARS is trained using the loss function below.

(3) $$LOSS_{MERS}=CrossEntrophy\left(s_{MARS},\hat{y}\right)+\alpha \|fc_{MERS}-fc_{FLOW}{\|}^{2}$$

where is a scalar weight that modifies how much motion features matter. MARS also distills the appearance as well as motion information.

### 2.3. Optimal Frame Selection

Frame Selection plays an important role in video classification accuracy. There are several basic conventional methods [42,43] for selecting the key-frames out of the video, such as: sequential comparison of frames, global comparison of frames, and the minimum correlation between frames [24]. Later [14] worked on detecting the key points for feature extraction via a Scale-Invariant Feature Transform (SIFT) [44] descriptor and pools the key points to find key-frames in videos. Bidirectional long short-term memory (Bi-LSTM) [27] was the first deep learning method introduced to automatically extract the highlights (key-frames) from videos. Afterward, Generative Adversarial Networks (GAN) [45] are used for keyframe detection in videos, which uses CNNs to extract the feature of each frame and then encodes the feature via LSTM.

## 3. Proposed Framework

**Figure 3:**
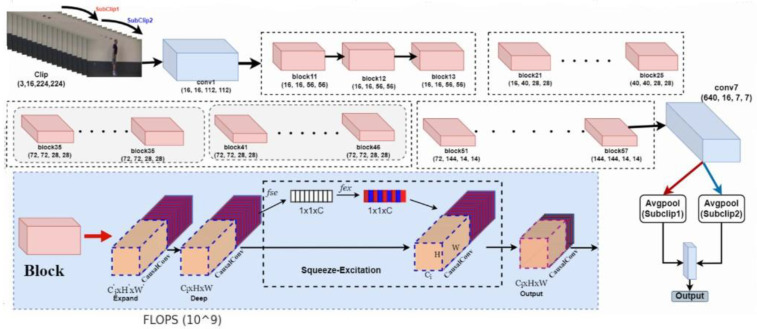
MoviNet-A2 with Causal Stream Buffer. Block layer contains many inner convolutional operations that are equal to the single red block (lock11, block12, …). The causal operation has been applied between the non-overlapping sub-clips to expand the receptive fields using the stream buffer concept. In each block layer, we have used the EvoNorm and Mish activation function to mitigate the generalization error [36].

This Section details our proposed framework, as shown in Figure 3.

### 3.1. Backbone

MoviNet, developed in 2020 by Facebook AI Research (FAIR) [46], is a highly efficient and scalable model, for mobile devices and other edge devices with limited computational resources. MoviNet’s architecture is based on a combination of depth wise and group convolutions, allowing it to achieve high accuracy while using fewer parameters and less computational power than other cutting-edge models. This makes it ideal for applications requiring real-time performance on mobile devices, such as video classification. The architecture search space has been designed to inspect how to unite operations of spatial, temporal, and spatiotemporal, such that Neural Architecture Search (NAS) can be better at finding the optimal feature combinations to trade-off efficiency and accuracy. MoviNet has used the same proposal generator as MobileNetV2; it reduces the size of the expansion layer and reduces the bottleneck in all blocks that share a similar size of bottleneck to maintain residual connections [47].

MoviNet A2 with Causal Stream Buffer is shown in Figure 3. The block layer contains many inner convolutional operations that are equal to the single red block (block11, block12, …). The causal operation is applied between the non-overlapping sub-clips to expand the receptive fields using the stream buffer concept. In each block layer, we used the EvoNorm and Mish activation function to mitigate the generalization error. The network prefers large bottleneck width multipliers in a range (2.5, 3.5), often shrinking or expanding them according to each layer. The balanced kernel size of 3 × 3 × 3 is used in this network, and the 5 × 3 × 3 kernel is used for the first down sampling layers at the later blocks. Nearly at every final stage, 1 × 5 × 5 spatial kernels are utilized to deal with the high-level features to benefit the classification from spatial features.

The Squeeze and Excitation (SE) [40] is used in the MobileNetv3 [36], MnasNet [48], and has also been utilized in this architecture. The structure of the SE block is depicted in Figure 3. The primary focus of the SE block is on reducing the number of parameters and operations. The SE block improves the quality of representations by modeling the inter-dependencies between the channels of its spatio-temporal features via 3D average pooling. The SE block is used in each sub-block as it is simple and computationally lightweight.

The transformed feature *X* of the Expand block is mapping the feature maps *D* deep block, where *DHWDC*. The features *D* are first passed through squeeze operations followed by the cumulative global overlapping pooling. The squeeze operations are used to extract the global information from each channel of the feature map, the cumulative global average pooling will reduce the *B H W D C* feature map to *B11DC*. Afterward, it goes through the excitation operation that is fully connected multi-layer perceptron (MLP). The MLP will produce the weights to adaptively scale each channel of the feature map. The architecture of MoviNet-A2 with Causal Stream Buffer is shown in Figure 3.

The tensor is then passed through the hard-sigmoid activation function. Particularly, element-wise multiplication is being performed during the scaling operation between the initial feature map and the output of hard sigmoid nonlinearity. Therefore, it is able to reduce the non-relevant information from the channels and the relevant information that does not produce more effect, which increases the representation power of the entire network. The other main ingredients of this architecture are the stream buffer and CausalConv [49].

### 3.2. Stream Buffers and Causal Convolution

The video contains the clips, and each clip carries the frames; some researchers prefer to use 16 frames for each clip, and some take 64 frames, as it drastically increases the accuracy, but leads to higher computational cost [36]. The Stream buffers process video in small consecutive sub-clips, which allow the stream buffer to store the features map from each sub-clip boundary to hold on with temporal dependencies, between the consecutive non-overlapping sub-clips. This helps MoviNet to reduce memory usage. In this architecture, the temporal convolution has been replaced with the causal convolution [49] that tends to make it unidirectional along the temporal dimension. In addition, the padding is computed to balance the convolution across all axes. Some paddings are merged after the final frames, which helps the stream buffer to carry out information to the frames of the next sub-clips, shown in Figure 3.

### 3.3. Generalization Gap

Normalization layers and activation functions play a crucial role in deep learning networks, ensuring stable optimization and enhanced generalization. A milestone in this field is the EvoNorm layer [17], which unifies normalization and activation layers, enabling them to generalize well throughout the entire architecture. This approach outperforms the traditional Batch Normalization (BN) and Rectified Linear Unit (ReLU) combination. EvoNorm has two variants: EvoNorm-B, which depends on the batch, and EvoNorm-S, which operates on individual samples. The EvoNorm-B0 variant relies on a single type of variance and combines two types of statistical moments in its denominator. It can be defined mathematically as follows:

(4) $$f\left(x\right)=\frac{x}{\sqrt{\left(s_{b,\omega ,h}^{2}\left(x\right)\right),\;v1x+s_{\omega ,h}^{2}\left(x\right)}}\;$$

where $s_{b,\omega ,h}^{2}\left(x\right)\;$ is instance variance, and $s_{\omega ,h}^{2}\left(x\right)\;$ is batch variance.

The EvoNorm-S0 variant incorporates the Sigmoid Linear Unit (SiLU) or Swish activation function, essentially a gated version of the sigmoid function. When parameter B becomes infinite, the Swish function behaves like a ReLU, while when it is zero, it acts as a linear function. The Swish activation function, proposed by Ramachandran et al. [18], is a smooth, non-monotonic function defined as:

(5) $$f\left(x\right)=x*sigmoid\;\left(\beta *x\right)\;$$

Here, *β* is a learnable parameter or a fixed constant. The smoothness of the Swish function has its advantages, such as fewer vanishing gradient issues and better performance in some cases compared to ReLU. However, this smoothness can also be a drawback when combined with dropout.

Dropout, introduced by Srivastava et al. [50], is a regularization technique that prevents overfitting in neural networks by randomly setting a fraction of input units to 0 during training at each update. This forces the network to learn more robust and generalized representations of the input data.

The smoothness and non-monotonic nature of the Swish function makes it more sensitive to the dropout rate. When dropout is applied, the activation function needs to be robust enough to handle the noise introduced by randomly dropping input units. Since the Swish function is smoother and more flexible than piecewise-linear functions like ReLU, it tends to be more sensitive to the dropout rate, leading to varying performance across different rates.

Due to these reasons, we used the Mish non-linearity [19] as a replacement for the Swish function:

(6) *f*(*β* ∗ *x*) = *x* ∗ tanh(*so f tplus* (*β* ∗ *x*))

Mish is unbounded above and bounded below, which helps it avoid saturation and provide strong regularization, addressing the overfitting issue. These properties make Mish a more suitable choice for working with different dropout rates.

The EvoNorm-S0 variant also has some characteristics of the group norm, such as dividing the post-activation features by the standard deviation, but here in this method, it is slightly different as it unifies the normalization and activation functions. EvoNorm-S0 with SiLU/Swish is written as:

(7) $$f\;\left(\beta *x\right)=\frac{x}{\sqrt{s_{\omega ,h,c}^{2}/g\left(x\right)}+\exp\left(\beta \;*\;x\right)}\;$$

where $s_{\omega ,h,c}^{2}/g\left(x\right)$ is channel variance. We obtained Equation (5) for EvoNorm-B0-Mish by combining Equations (2) and (3).

(8) $$f\;\left(\beta \;*\;x\right)=\frac{x\;*\;\tan h\left(softplus\left(\beta \;*\;x\right)\right)\;}{\sqrt{\left(s_{b,\omega ,h}^{2}\left(x\right)\right),\;v1x+s_{\omega ,h}^{2}\left(x\right)}}\;$$

We obtained Equation (6) for EvoNorm-S0-Mish by combining Equations (3) and (4).

(9) $$f\;\left(\beta *x\right)=\frac{x\;*\;\tan h\left(softplus\left(\beta \;*\;x\right)\right)}{\sqrt{s_{\omega ,h,c}^{2}/g\left(x\right)}}\;$$

### 3.4. Optimal Frame Selection Using Optical Flow

Optical flow is the apparent motion of objects, surfaces, and edges in a scene caused by the relative motion between an observer and the scene. It is used to estimate the motion between consecutive frames in a video sequence, which can be useful in various computer vision tasks such as video processing, object tracking, and motion analysis. The motion in the $\left(x,y\right)\;$spatial domain is determined as a function of time (*t*) In the first frame, the image intensity at a given point $\left(x,y\right)\;$with respect to time is represented as $I\left(x,y,t\right)$. When the pixels at this point move by dx and dy over time “t” in the next frame, a new image is obtained:

(10) $$I\left(x,y,t\right)=I\left(x+dx,y+dy,t+dt\right)\;$$

In consecutive frames, the rate of change of pixels may be minimal, indicating that the frame contains similar contextual information as the previous frame. If consecutive sets of frames are taken, there is a chance of obtaining frames with similar contextual information. Feeding a model with similar information can harm its performance as it is trained with distinct kinds of visual information. Therefore, it is essential to find the key-frames containing distinct information.

To calculate optical flow, various methods have been proposed in the literature [51]. In this work, the TVL1 method is utilized to extract optical frames. By applying the Taylor Series approximation, an expanded equation is obtained:

(11) $$I\left(x,y,t\right)=I\left(x,y,t\right)+\frac{\partial I}{\partial x}dx+\frac{\partial I}{\partial y}dy+\frac{\partial I}{\partial t}dt$$

Then, both equations will give us the optical flow constraint equation:

(12) $$I_{x}*\mu_{x}+I_{y}*\mu_{y}+I_{t}$$

Here, *I_x_* and *I_y_* represent the change in image intensity in the *x* and *y* directions for adjacent pixels in the same image, and It represents the change in intensity of the same pixel at adjacent moments. If the change in image intensity in consecutive frames is close to zero, it means that the frames have similar information and are not key-frames.

To find the change in image intensity, the pixel distribution in the image must be determined. In our approach, we incorporated the Local Binary Pattern (LBP) to capture local texture information in images. LBP is a powerful texture descriptor that examines the relationship between a pixel and its neighbors to encode the local structure within an image. By computing the LBP for each pixel, we were able to create a histogram representing the local texture patterns present in the image.

To provide a more comprehensive representation of the image content, we combined the LBP histogram with the original pixel intensity histogram. This joint histogram encapsulates both texture and intensity information, offering a richer understanding of the image’s features. The combination of these two histograms allows us to more accurately identify key-frames with distinct visual information, as shown in Figure 1.

These advanced techniques enable better frame selection compared to random selection, ensuring that the model is exposed to a diverse set of visual information for improved performance. By leveraging the complementary information provided by the LBP and pixel intensity histograms, our method effectively distinguishes between key-frames and normal frames, resulting in a more robust model capable of handling a wider range of visual input. In future work, we plan to use convolutional neural networks (CNNs) for optical flow estimation to improve key-frame identification. We also aim to incorporate advanced feature extraction methods, such as Scale-Invariant Feature Transform (SIFT) and Speeded-Up Robust Features (SURF), for more accurate and reliable key-frame selection.

## 4. Datasets and Metrics

We focused on two benchmark datasets that contain a medium amount of data: UCF101 [13] and HMDB51 [14]. The UCF101 dataset contains the 101 action classes with a total of 13,320 videos of 25 fps and has three split tests/trains. We used the first split of UCF101 for our proposed model. We conducted a comparison in Table 1 and Table 2 through an ablation study. Table 3 presents a comparison of EMO-MoviNet and its variants. In this study, we substituted Swish with Mish and optimal frame selection with random frame selection, all within the context of the initial 20 classes. Conversely, when comparing in Table 4, we extended our analysis to the entire UCF101 dataset. HMDB51 contains a large collection of realistic videos of different actions with challenging background, occlusions, deformation, and illumination conditions. The dataset is constituted of 6849 video clips from 51 action groups. Each category possesses at least 101 clips. It has three training/testing slips. In each split, each action has 70 clips for training and 30 clips for testing. We denoted the first split of the UCF101 and HMDB51 as UCF101-1 and HMDB51-1. We have taken the initial 20 classes out of the UCF101 classes for the experiment shown in Table 1.

### Metrics

For the UCF101 dataset, we report the Top-1 accuracy as a metric of split 01. For the HMDB51, we report the average accuracy of all three splits.

## 5. Experiments and Analysis

In this section, we discuss all the experiments that we conducted to test all amendments in the MoviNet and the optimal frame selection.

### 5.1. Training Details

The model is trained on GeForce GTX 3080, and we used the momentum SGD with weight decay 0.00001 and momentum 0.9. The video is divided into clips that contain 16, 64, and 128 frames for UCF101. For the 16-frame clip, we kept the batch size of 12. We trained the EMO-MoviNets model on the UCF101 and HMDB51 fine-tuned from the pre-trained model of the Kinetic dataset. We experimented with training without a pre-trained model as well, which gives less accuracy and also takes as much time to train as the random weights. The learning rate is 0.001 initially and then after every 8k iterations, the learning rate decreases by 10. We extracted frames at 25 fps for UCF101 and resized them to 256 pixels. We experimented with the Adam optimizer as well; it was able to learn the weights in fewer epochs than the SGD, but it ended with the overfitting. We also used some regularization and data augmentation techniques to reduce overfitting. However, these techniques were not giving the desired results. Therefore, we used the SGD optimizer. During training, the 256 resized frames were randomly cropped to the 224; we also applied the random horizontal flip and multiscale corner crop, and we subtracted the Activity means from RGB inputs.

### 5.2. Inference

At the test time, we used the center crop and average crop for all the clips in both UCF101 and HMDB51. We used the average accuracy of all three splits in the HMDB51 to compare with the recent work.

## 6. Results and Discussion

We begin with the analysis of the impact of different amendments in the architecture on the initial 20 classes of the UCF101 dataset, and the results are shown in Table 1. Next, we compared the computational efficiency with the motion representation methods on both datasets shown in Figure 2. In HMDB51, our model has outperformed all methods in both accuracy and computational complexity by a sufficient margin. Whereas, in UCF101 our model is giving fewer FLOPS and parameters at the cost of 12% accuracy.

### 6.1. Discussion

Our study emphasizes the significance of normalization layers and activation functions in deep learning networks for stable optimization and enhanced generalization. We discuss the innovative EvoNorm layer, which outperforms the traditional Batch Normalization and Rectified Linear Unit (ReLU) combination. Two EvoNorm variants, EvoNorm-B and EvoNorm-S, are presented, with the latter incorporating the Swish activation function.

Furthermore, our method’s superior performance could be attributed to a variety of factors. First, our novel changes may have better captured the intrinsic characteristics of the HMDB51 and UCF101 datasets. Second, our method may be more robust to overfitting, a common issue in machine learning, especially when dealing with high-dimensional data. Lastly, the reduction in computational cost suggests that our method may be more computationally efficient, which could be due to a more effective algorithm design or more efficient use of computational resources.

#### 6.1.1. Adopting Mish Activation Function within EvoNorm: Boosting Network Performance by Replacing Swish

The smoothness of the Swish function is identified as a drawback when combined with dropout, as it is more sensitive to the dropout rate. To address this issue, we suggest using the Mish non-linearity as an alternative, as it is better suited for working with different dropout rates. Mish avoids saturation and provides strong regularization, making it a more robust and adaptable activation function, ultimately improving deep learning network performance.

We provide empirical evidence to support our claims by presenting a table comparing the performance of two different activation functions, EvoNorm-swish and EvoNorm-mish, within the same deep learning network architecture, EMO-MoviNet A2*. Table 1 displays the accuracy achieved by each model.

#### 6.1.2. Combining TVL1 and Local Binary Patterns for Precise Key-Frame Identification in Optical Flow

The significance of optical flow in computer vision tasks is essential for estimating motion between consecutive frames in a video sequence. This information can be utilized for various applications, such as video processing, object tracking, and motion analysis. To enhance model performance, it is crucial to identify key-frames containing distinct visual information within the video sequence.

Our proposed method employs the TVL1 approach for extracting optical frames and the Local Binary Pattern (LBP) technique for capturing local texture information. By merging the LBP histogram with the original pixel intensity histogram, the method offers a more comprehensive representation of the image’s features, ultimately enabling more precise key-frame identification.

To demonstrate the effectiveness of this approach, we provide a comparison in Table 2 comparing the proposed method with random frame selection.

### 6.2. Limitations

A major limitation of this study is the need to validate the proposed method on larger datasets, such as the Kinetics dataset, which requires high computational cost. However, this limitation also illustrates an important consideration in algorithm development, as methods must strike a balance between accuracy and computational efficiency.

### 6.3. Results

In this study, we evaluated contemporary action recognition methods on two well-known datasets, UCF101 and HMDB51. The results of this comparison are presented in Table 3. In order to ensure a fair and comprehensive comparison, we took into consideration crucial factors, including the pre-trained dataset, GFLOPS (Giga Floating Point Operations Per Second), and the total number of parameters involved in the model.

**Table 3:** The accuracy and GFlops of EMO-MoviNet variants (A0–A2) on HMDB51-1 dataset. We implemented the EMO-MoviNet variants with stream buffer (A0*, A1*, A2*) and without stream buffer (A0, A1, A2). The bold text shows the performance improvements.

Model	Accuracy	GFlops	Parameters
EMO-MoviNet-A0	71.39	**2.71 G**	3.1 M
EMO-MoviNet-A0*	**74.83**	2.73 G	**4.6 M**
RNxt101-R [52]	63.8	76.68 G	47.6 M
RNxt101-F [52]	71.2	67.88 G	47.6 M
**EMO-MoviNet-A1**	74.38	**6.022 G**	4.6 M
EMO-MoviNet-A1*	**77.42**	6.06 G	**4.6 M**
MERS	71.8	6.68 G	47.6 M
MERS-R	72.9	76.88 G	47.6 M
MERS-F	72.4	67.88	47.6 M
MERS-R + F	74.5	-	47.6 M
**EMO-MoviNet-A2**	79.36	**10.3 G**	4.8 M
EMO-MoviNet-A2*	**80.6**	10.4 G	**4.8 M**
MARS	72.8	76.68 G	47.6 M
MARS-R	73.1	76.88 G	47.6 M
MARS-F	74.5	67.88	47.6 M
MERS-R + F	75	-	47.6 M

#### 6.3.1. EMO-MoviNet-A2 (20 Classes-UCF101)

In our study, we implemented EMO-MoviNet-A2 with modifications to the architecture and optimal frame selection, as illustrated in Table 2. Due to limited resources, we evaluated our model on the initial 20 classes of the UCF101 dataset. The optimal frame selection yielded superior results compared to random selection, as the latter often leads to the model learning similar contextual information, resulting in unfamiliarity with other information. As shown in Table 2, our frame selection method improved accuracy by 0.4–0.7% with fewer epochs than random selection.

Additionally, we incorporated the self-regularized non-monotonic activation function, Mish, which, with the same computational expense and time, improved accuracy by 0.8–1% in fewer epochs while maintaining constant network parameters and hyperparameters. Mish outperforms Swish due to two factors: it is unbounded above, avoiding saturation and slow training caused by near-zero gradients, and bounded below, offering strong regularization and reducing overfitting. Furthermore, Evolving normalization provided a 1.2–1.5% accuracy improvement compared to methods utilizing normalization and activation functions separately, as demonstrated in Table 3.

#### 6.3.2. EMO-MOVINET (A0–A2)

We compared the recent action recognition methods on the UCF101 and HMDB51 dataset and report the result in Table 3. For a fair comparison, we enlisted the important factors such as pre-trained dataset, GFLOPS, and parameters.

MoviNet [3] introduced a series of MoviNet models, with seven variants (A0–A6) designed for the Kinetics 600 dataset without the use of stream buffers and three additional variants (A0–A2) that incorporated stream buffers. As the variants progressed from A0 to A6, both resolution and depth scaling increased, resulting in enhanced computational power. This increase in computational power was accompanied by a corresponding improvement in the model’s overall accuracy, as demonstrated in Table 3.

We proposed the modified EMO-MoviNet-A2* architecture that integrates EvoNorm, Mish activation, and optimal frame selection to improve the accuracy and efficiency of action recognition. The asterisk notation indicates that this model also incorporates the stream buffer concept. The EMO-MoviNet-A2* model successfully attained satisfactory levels of accuracy on both the UCF101 and HMDB51 datasets. Simultaneously, the model maintained lower computational costs and a reduced number of operations, as depicted in Figure 2. To achieve these outcomes, we made adjustments to the model’s architecture, including increasing its depth and resolution.

These modifications resulted in an elevated number of operations, which consequently led to an increase in the model’s overall computational energy requirements. Given the impressive performance of the A2* variant in balancing accuracy and computational demands, we chose to concentrate exclusively on this particular model for our research. Accordingly, we have modified the EMO-MoviNet-A2* architecture based on the MoviNet-A2* model.

Our proposed model, EMO-MoviNet-A2*, achieved a remarkable 91.83% accuracy on the UCF101 dataset, as shown in Table 4, demonstrating its high efficiency and effectiveness. This performance was attained with lower Floating Point Operations Per Second (FLOPS) and fewer operations and parameters compared to the MARS [10] model. Specifically, FLOPS decreased by 86%, and the number of parameters was reduced by 90%. It is important to note that the model’s accuracy could potentially be improved further by using 64 clips, as is done in MARS. However, this approach would have a significant impact on training time. On the HMDB51 dataset, our model achieved an accuracy of 81.53%, which is 4–6% higher than MARS [10], while maintaining 86% lower FLOPS and 90% fewer parameters, as shown in Table 3.

State-of-the-art results can be produced by combining RGB and flow streams in a 3D CNN, as the model benefits from both appearance and explicit information. However, calculating optical flow for multiple datasets can be computationally expensive, often requiring hundreds of optimization iterations per frame. This process necessitates the learning of two separate CNN streams, leading to increased computational costs and a higher number of parameters for the model to learn. Despite this, the model can achieve improved accuracy as a result.

In our study, we compared the results with the RGB stream alone and found that our method, which relies solely on the explicit information derived from the RGB stream, yielded higher accuracy than techniques employing a two-stream approach that leverages information related to motion. We implemented several MoviNet variants, as shown in Table 3. The EMO-MoviNet-A2 (w/o stream buffer) model achieved 89.7% accuracy on the UCF101 dataset and 79.3% on the HMDB51 dataset, with nearly identical FLOPS and the number of parameters compared to the EMO-MoviNet-A2* (with stream buffer) model. This further demonstrates the effectiveness of our proposed model and its potential for application in action recognition tasks.

Table 3 presents a comprehensive comparison of the performance of three EMO-MoviNet variants on the HMDB51 dataset, alongside the results obtained when incorporating the proposed EMO technique. In this table, the stream buffer is indicated by an asterisk. Notably, the EMO-MoviNet-A0* variant demonstrates almost identical GFLOPs as the original EMO-MoviNet-A0, while achieving a 4% increase in accuracy. This variant surpasses the RNxt101-R in terms of accuracy by 8%. Table 4 presents the result of the handcrafted techniques and some of the recent work, in which our proposed model performed well on both UCF101 and HMDB51 dataset.

Similarly, the EMO-MoviNet-A1* variant exhibits nearly equal GFLOPs when compared to EMO-MoviNet-A1, yet boasts a 4% higher accuracy than EMO-MoviNet-A1 and a 6% higher accuracy than MERS-R. By integrating the EvoNorm layer, Mish activation function, and optimal frame selection technique, the EMO-MoviNet-A0* and EMO-MoviNet-A1* models outperform their respective EMO-MoviNet-A1* and EMO-MoviNet-A2* counterparts.

**Table 4:** Top-1 accuracy of the proposed model on Kinetics 600 on UCF101 and HMDB51 dataset. Methods with initializing weights with Kinetics pre-trained one. Our method has beat the accuracy of the above network in both datasets. Our proposed method has outperformed many recent works in accuracy with lower computational expense, number of parameters, and operations (MADDS) in UCF101 and HMDB51 datasets. The bold text shows the performance improvements.

Model	Pre-Trained	UCF101	HMDB51
IDT [28]	-	86.4%	61.7%
C3D + IDT	Sports1M	90.4%	-
TDD + IDT	-	91.5%	65.9
DIN + IDTD	-	89.1%	65.2%
ResNext + RGB	Kinetics	91.7%	75.5%
C3D	Sports1M	90.4%	65.4%
Two-stream [6]	Imagenet	88.0%	59.4%
ActionFlowNet	-	83.9%	56.4%
**EMO-MoviNet-A2**	Kintics600	89.7%	79.3%
EMO-MoviNet-A2*	Kintics600	**91.8**%	**81.53**%

In recent years, numerous researchers [18,19,23,24] have employed pre-trained models based on multiple datasets, such as ImageNet, Kinetics 600, and Kinetics 700, to achieve higher accuracy in their respective models. This approach is effective because the models become familiar with the feature information of a wide variety of classes, thereby improving their performance on datasets containing fewer classes.

For instance, D3D [53], when pre-trained on Kinetics-400, demonstrated lower accuracy compared to the models pre-trained on Kinetics-600. In our study, we opted to use a pre-trained model based on Kinetics-600, which led to an increase in accuracy of approximately 3–4%. Our model achieved an accuracy of 81.53% on the HMDB51 dataset, which is 5–7% higher than that of the MARS model [10], as presented in Table 5.

Additionally, our model has the potential to surpass the state-of-the-art networks by incorporating a two-stream technique or distillation. However, these approaches can be time-consuming and require substantial computational resources. Nonetheless, the use of pre-trained models on multiple datasets proves to be a promising method for enhancing the accuracy and overall performance of the model in the context of our research.

This comprehensive analysis highlights the effectiveness of incorporating the proposed EMO technique, which combines EvoNorm, Mish activation function, and optimal frame selection, in enhancing the accuracy of EMO-MoviNet variants while maintaining comparable computational efficiency.

## 7. Conclusions

In this study, we designed the EMO-MoviNet network, pre-trained on Kinetics, and evaluated its performance using the UCF101 and HMDB51 datasets. To address the generalization gap between training and testing, we implemented EvoNorm (Unified norm-activation) with S and B variants, combined with Swish and Mish activation functions. By selecting the most contextually informative frame from each video, we optimized the input for our model. Comparisons with recent work revealed that our EMO-MoviNet outperformed other networks on both datasets, achieving improved accuracy while requiring approximately three times fewer floating-point operations and six times fewer parameters. Moreover, our study employs conventional techniques for selecting key frames from video footage. Moving forward, we intend to leverage convolutional neural networks (CNNs) to enhance optical flow estimation, thereby refining our key-frame identification process. Additionally, we have aspirations to integrate sophisticated feature extraction approaches like the Scale-Invariant Feature Transform (SIFT) and Speeded-Up Robust Features (SURF). These endeavors are aimed at achieving greater precision and reliability in the selection of key frames.

## Figures and Tables

**Table 1:** Top-1 accuracy of the proposed model on UCF101-20 Classes dataset. The table demonstrates the effectiveness of the proposed Mish non-linearity as an alternative to the Swish function. By comparing the accuracy of both models, the reader can observe the impact of using the Mish activation function on the overall performance of the MoviNet A2* architecture. The bold text shows the increase in accuracy.

Model/Activation	Accuracy
MoviNet-A2* Swish	87.52%
MoviNet-A2* Mish	**88.11%**

**Table 2:** Top-1 accuracy of the proposed model on UCF101-20 Classes dataset. The table showcases the superiority of the proposed method in selecting key-frames containing distinct visual information, which translates to enhanced model performance. This improvement is attributed to the synergistic combination of LBP and pixel intensity histograms, which allows the method to effectively differentiate between key-frames and normal frames. Consequently, the developed model is more robust and better equipped to process a diverse range of visual inputs. The bold text shows the increase in accuracy, due to optimal frame selection.

Model/Activation	Pre-Trained	Accuracy
MoviNet A2* Random Frame Selection	Kinetic-700	87.25%
MoviNet-A2* **Optimal Frame Selection**	Kinetic-700	**87.76%**

**Table 5:** Gflops and parameters on UCF101 and HMDB51 dataset. We measure the total Gflops per video across all frames. In the HMDB51 dataset, our method has outperformed all the above networks with a lower computational expense. The * symbol denotes that the stream buffer and causal convolution is used in the network. The bold text shows the performance improvements.

Model	Pre-Trained	UCF101	HMDB51	GFlops	Parameters
STAM16 [12]	-	97%	-	270 G	96 M
I3D [9]	Imagenet-kinetics	95.6	74.8	108 G	28 M
S3D [54]	Imagenet-kinetics	96.8	75.9	66.4 G	-
R(2 + 1)D [33]	Sports1M	93.6%	66.6%	152 G	-
Artnet [55]	Kinetics600	**94.3%**	70.9%	-	-
FASTER32 [56]	Kinetics600	96.9%	75.7%	67.7 G	-
TSM-R50 [57]	-	95.9%	73.5%	65 G	24.3 M
STM-R50 [58]	-	96.2%	72.2%	67 G	24 M
MsNet-R50 [59]	-	-	77.4%	67.9 G	24.6 M
VidTr-L [60]	-	96.7%	74.4%	59 G	-
MARS-RGB [10]	Kinetics600	94.6%	76.68G	47.63 M	
MARS-Flow [10]	Kinetics600	95.6%	74.5%	66.88 G	-
EMO-MoviNet-A2	Kintics600	89.7%	79.3%	10.4 G	4.8 M
EMO-MoviNet-A2 *	Kintics600	91.8%	**81.53**%	**10.3 G**	**4.8 M**

## Data Availability

Not applicable.
